# Biological Evaluation and 3D-QSAR Studies of Curcumin Analogues as Aldehyde Dehydrogenase 1 Inhibitors

**DOI:** 10.3390/ijms15058795

**Published:** 2014-05-16

**Authors:** Hui Wang, Zhiyun Du, Changyuan Zhang, Zhikai Tang, Yan He, Qiuyan Zhang, Jun Zhao, Xi Zheng

**Affiliations:** 1 School of Light Industry and Chemical Engineering, Guangdong University of Technology, Guangzhou 510500, China; E-Mails: wanghuibetty1988@126.com (H.W.); changyuanzhang@126.com (C.Z.); tzkcarlos@163.com (Z.T.); hxg_129@126.com (Y.H.); fumaihuanxue@126.com (Q.Z.); junzhao60@hotmail.com (J.Z.); zhengxi08@hotmail.com (X.Z.); 2 Guangzhou Improve Medical Technology Co., Ltd., Guangzhou 510530, China; 3 Susan Lehman Cullman Laboratory for Cancer Research Ernest Mario School of Pharmacy, Rutgers, the State University of New Jersey, Piscataway, NJ 08854, USA

**Keywords:** curcumin, curcumin analogues, ALDH1, inhibitor, 3D-QSAR

## Abstract

Aldehyde dehydrogenase 1 (ALDH1) is reported as a biomarker for identifying some cancer stem cells, and down-regulation or inhibition of the enzyme can be effective in anti-drug resistance and a potent therapeutic for some tumours. In this paper, the inhibitory activity, mechanism mode, molecular docking and 3D-QSAR (three-dimensional quantitative structure activity relationship) of curcumin analogues (CAs) against ALDH1 were studied. Results demonstrated that curcumin and CAs possessed potent inhibitory activity against ALDH1, and the CAs compound with *ortho* di-hydroxyl groups showed the most potent inhibitory activity. This study indicates that CAs may represent a new class of ALDH1 inhibitor.

## 1. Introduction

The aldehyde dehydrogenase 1 (ALDH1) family is mainly present in the cytoplasm of various tissues and catalyzes the oxidation of aliphatic and aromatic aldehydes to the corresponding carboxylic acids in the presence of NAD or NADP as cofactor [1]. It plays an important role in the detoxification of peroxidic aldehydes produced by ultraviolet light absorption, protecting the lens of the eye [2]. Recently, the role of ALDH1 in drug resistance was observed in the case of cyclophosphamide chemotherapy of cancer cells with high level of expression of ALDH1 [3,4]. It is reported that the oxidation of aldophamide could be accomplished directly by ALDH1, and downregulation of ALDH1 by antisense RNA could result in increasing the sensitivity of tumour cells to 4-hydroperoxy-cyclophosphamide (4-HC), an active derivative of cyclophosphamide [5]. Decreasing the ALDH1 protein level or blocking the enzyme activity led to an increase in the sensitivity of chemotherapy [6]. It was also demonstrated that ALDH1 activity was related to metastatic potential in murine OS cells [7,8] and ALDH1 inhibitors induced apoptosis in the lymphoid cell line BAF_3_H_16_ over expressing the *bcl2* gene [9–11]. Therefore, inhibiting ALDH1 activity in tumor cells may be a strategy to alleviate chemoresistance and induce apoptosis in some cancer cells.

Curcumin is a natural occurred compound which is extracted from *rhizome* of *Zingiberaceae Turmeric*. Curcumin has shown antioxidant, anti-inflammatory, antiviral, antibacterial, antifungal and anticancer activities [12]. Our research group reported that curcumin and its analogues synthesized in our laboratory [13] possessed potent inhibitory activities on PC-3, Panc-1, and HT-29 cells, which have high expression of ALDH [14]. These suggest that curcumin analogues (CAs) may serve as a new class of ALDH1 inhibitors. In this paper, the inhibitory activity, mechanism mode, molecular docking and 3D-QSAR of curcumin analogues against ALDH1 were studied.

## 2. Results and Discussion

### 2.1. Activity Assay

The structure and activity of CAs data were showed in Table 1. Analysis of structure activity relationship indicated that the change of glutaric enone and phenyl substituents had important influence on the activity of the compounds. The activity data pIC_50_ is range 4.13 to 5.46. Comparing six different glutaric ketones, the CAs have a higher activity when the X group is cyclopentanone. When the glutaric enone is the cyclopentanone and the phenyl substituent is an electron-withdrawing group, the CAs have a higher activity. At the same time, the curcumin and disulfiram, which to ALDH1 inhibitory activity, show the data IC_50_ values of 36.9 and 2.91 μmol/L, respectively. The activity of compound **6** (IC_50_ 3.41 μmol/L) is over 10 times higher than that of curcumin and similar to disufiram. Therefore, curcumin analogues may serve as a new class of ALDH1 inhibitors.

### 2.2. Kinetic Analysis of Selected Compounds on ALDH1

**Figure 1.**
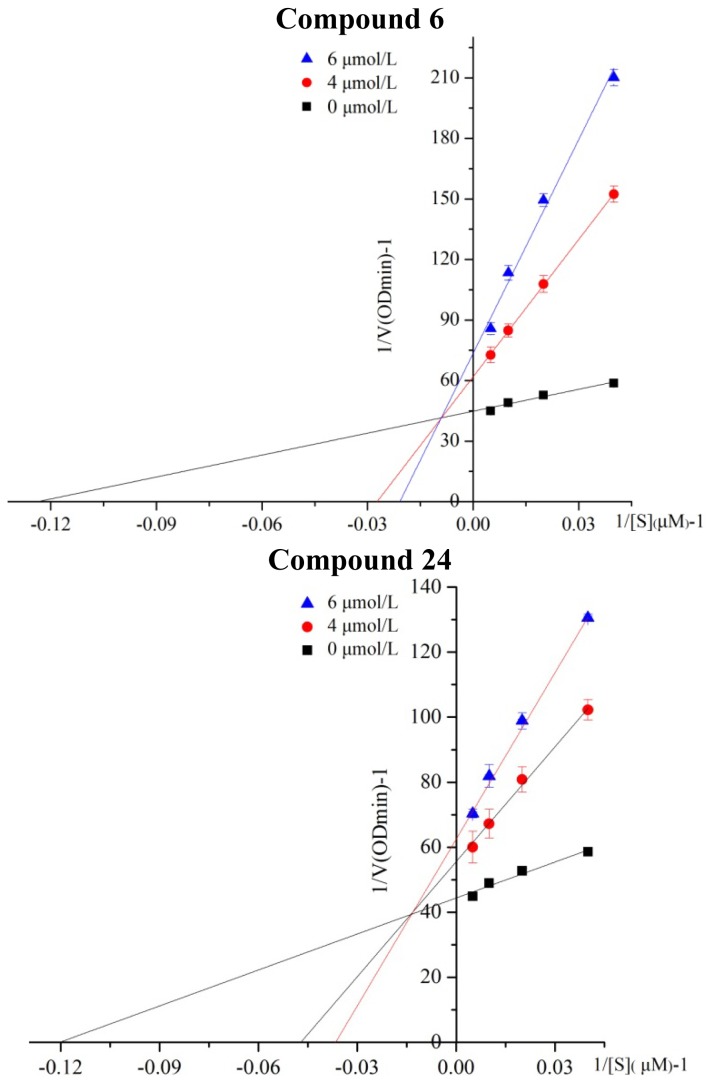
Lineweaver–Burk plots for inhibition of compound **6** and compound **24** against aldehyde dehydrogenase 1 (ALDH1) for the catalysis of propanal.

The inhibitory mechanisms of selected compound **6** and **24** against ALDH1 during the oxidation of proponal were determined by the same methods. Double-reciprocal plots of the inhibition kinetics of selected compounds against ALDH1 are shown in Figure 1. Compound **6** and **24** both were mixed-competitive inhibition type, as illustrated in Figure 1. The Michaelis constant (km) of Compound **6** and **24** is 36.6 and 24.5 μM, respectively.

### 2.3. CoMFA and CoSIA Statistical Results

It is well known that the CoMFA and CoMSIA models are alignment sensitive, and the quality and the predictive ability of the models are directly dependent on the alignment rules. 3D-QSAR model with a *q*^2^ value > 0.5 and *r*^2^ > 0.9 are considered statistically significant and highly self-consistent, respectively. The statistical results of CoMFA and CoMSIA are shown in Table 2. The optimal number of components depend on selecting the highest *q*^2^ value. By PLS analysis result a high *q*^2^ value of 0.606 with 9 components for CoMFA. The non-cross-validated PLS analysis results in a conventional *r*^2^ 0.999; *F* 2577.847 and a standard error of estimation (*SEE*) of 0.011, the steric and electrostatic contributions were found to be 55.2% and 44.6% respectively.

Table 2, shows the PLS results of CoMSIA analysis using different combinations. The SED field descriptors exhibited highest *q*^2^, better *SEE* and *F* values than the others. Therefore combinations of steric (S), electrostatic (E), and hydrogen bond donor (D) fields was selected as the best model. The CoMSIA model *q*^2^ of 0.56 with an optimized component number of 6 with a low *SEE* of 0.031 and *F* value of 210.105. The steric, electrostatic contributions and hydrogen bond donor were found to be 28.7%, 37.8% and 33.8%.

**Figure 2.**
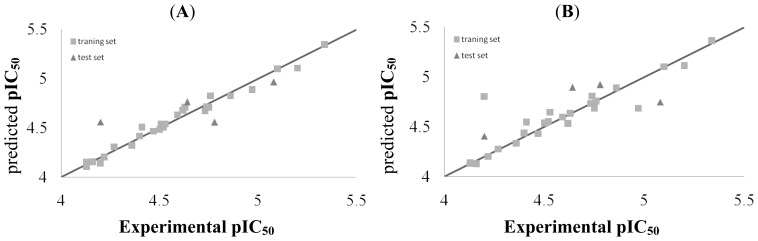
(**A**) The experimental and Predicted activities of CoMFA; (**B**) The experimental and Predicted activities of CoMSIA.

The ultimate characteristic of the 3D-QSAR technique is the validation of the externally driven 3D-QSAR model by means of calculating quantitatively the activities of test set compounds. The predicted activities for the inhibitor versus their experimental activities are listed in Table 3. Test sets are generally used to evaluate the external predictive capabilities of QSAR models. The correlation between predicted activities and the experimental activities of CoMFA and CoMSIA model is plotted in Figure 2. It is good linear relationship between the predicted and experiment activities of the dataset. Among them, compound **24** is found to be an outlier with residual values of 0.115 and 0.334 for CoMFA and CoMSIA model, respectively. There are numerous reasons for the presence of outliers, such as incorrectly experimental values or non-representative sampling designs. The solubility of compound **24** is not good, the errors may be relatively large.

### 2.4. Contour Maps Analysis

#### 2.4.1. CoMFA Contour Maps

**Figure 3.**
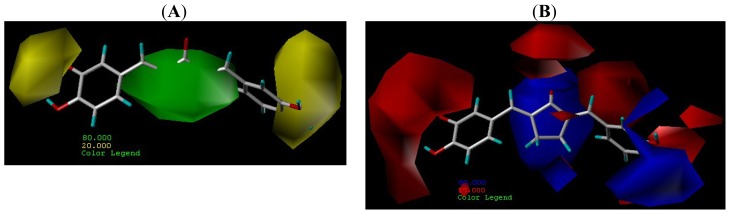
CoMFA steric filed (**A**) and electrostatic field (**B**). S fields: favored (green) and disfavored (yellow); E fields: electropositive (blue) and electronegative (red).

CoMFA steric and electrostatic contour maps are shown on compound **6** (the highest inhibitory activity) as the template. The steric fields are represented by green and yellow colored contours, in which green areas indicate regions where increased steric hindrance would increase the ALDH1 inhibitory activity, whereas the yellow areas suggest regions where the bulky groups are not favored. From the Figure 3A static fields, a large green contour overlaps the substituent group of R1 position that illustrates increasing bulky substituent is helpful to increase activity of inhibitors, as compounds **18**, **19**, **20** have more potent inhibitory activity than compound **17**.

The electrosteric fields are represented by blue and red contours and depict the position where positively charged groups and negatively charged groups would be beneficial to the inhibitory activities, respectively. From the Figure 3B electrostatic field, near the R1 substituent and phenyl ring 2 area M2 is a large blue contour respectively that ring M2 area indicates an increasing inhibitory negatively charged group. A large red contour near the phenyl indicates positive charged groups increasing inhibitory activity and compounds **18**, **19**, **20** show more potent inhibitory activity than compound **21**.

#### 2.4.2. CoMSIA Contour Map

**Figure 4.**
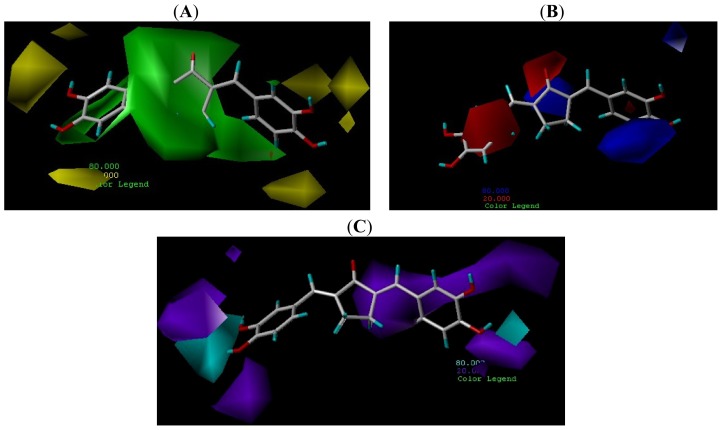
CoMSIA steric filed (**A**) and electrostatic field (**B**); CoMSIA Hydrogen bond donor (**C**). S fields: favored (green) and disfavored (yellow); E fields: Lipophilic (blue) and hydrophobic (red); D field: favored (purple) and disfavored (cyan).

CoMSIA steric, electrostatic and hydrogen-bond donor contour maps are shown also on compound **6** (the highest inhibitory activity) as the template. From the Figure 4A static and Figure 4B electrostatic fields, the distribution of the area M1, area M2 and area M3 are almost consistent with the CoMFA models. In the hydrogen-bond donor field, the cyan contours, represent the hydrogen bond-donating groups increasing the activity, the purple contour decreasing the activity. From the Figure 4C, the cyan contours are near the phenyl ring hydroxyl groups. Introducing hydroxyl groups on the phenyl ring, which can improve activity.

### 2.5. Binding Model Analysis

**Figure 5.**
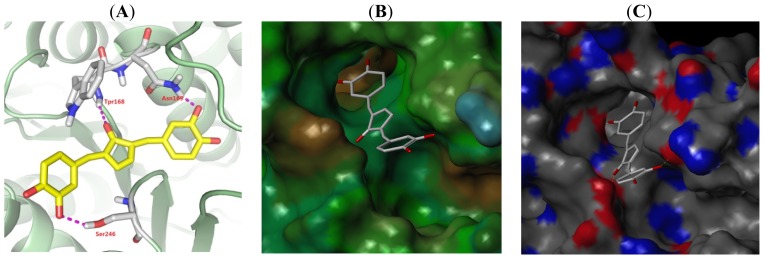
The binding mode between compound **6** with ALDH1 (**A**). Active site MOLCAD surface representation Liphilic potential (**B**) and Hydrogen Bonding (**C**); (**B**) Brown: Hydrogen and green: Hydrophlic; (**C**) Red: Hydrogen donor and blue: Hydrogen acceptor.

In order to clarify the combination of CAs with ALDH1 and determine the stability of the 3D-QASR modes, selecting the most active compound 6 with ALDH1 for Surflex-Dock. The Surflex-Dock score of compound 6 is 7.34 and that score showed that the *in vitro* tested result is consistent with the molecular docking. Figure 5A is the binding mode of A2 with active sites of ALDH1. Compound 6 was mainly surrounded by active pocket included in the residues of Cys301, Ile303, Gly245, Thr244, Phe243, Asn169, Trp168 and so on. Compound 6 carbonyl O and OH respectively formed hydrogen bond with NH_2_ of Tpr168 (Å 2.511) and NH_2_ of Asn 169 (Å 2.208) located inside the pocket, which has important inhibitory activity towards ALDH1. Besides compound 6 also formed hydrogen bond with Ser246 (Å 2.220) outside the activity pocket. Trp168 is important to form a π bond with compound 6 glutaric enone. From the Figure 5B, MOLCAD lipophilic potential (LP) showed that the glutaric enone (area M1) and phenyl ring 2 (area M2) are closed to the hydrophobic region and indicate increased hydrophobic group favor to improve inhibitory activity. This conclusion is consistent with the CoMSIA hydrophobic contour group. From the Figure 5C MOLCAD hydrogen bonding sites of the binding surfaces, the hydrophobic pocket has presented several hydrogen donors and acceptors. While the compound 6 formed three hydrogen bonds just as an acceptor, increasing the inhibitor hydrogen donor may strengthen the inhibitory activity.

**Figure 6.**
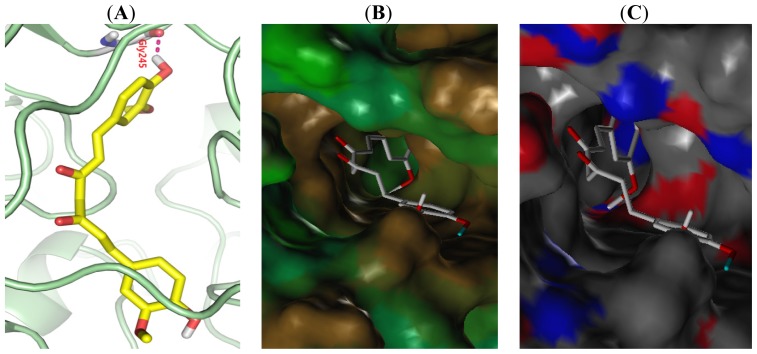
The binding mode between curcumin with ALDH1(A). Active site MOLCAD surface representation Liphilic potential and Hydrogen Bonding; (**B**) Brown: Hydrogen and green: Hydrophlic; (**C**) Red: Hydrogen donor and blue: Hydrogen acceptor).

Figure 6A is the binding mode of curcumin with active sites of ALDH1. Although curcumin is able to deep into the pocket, which just formed hydrogen with Gly245 outside the pocket, and the collision is very high. From the active site MOLCAD surface representation Liphilic potential and Hydrogen Bonding, we find that curcumin can not form hydrogen bonds in the active pocket and the skeleton diphenyl ketone of curcumin is too large, which is unfavorable combination with ALDH1. According to molecular docking and 3D-QSAR, a series of novel derivatives were designed. The activities of newly designed virtual molecules were predicted using CoMFA, CoMSIA models and the results were shown in Table 4.

## 3. Materials and Methods

### 3.1. Materials

Aldehyde dehydrogenase, NAD, dimethyl sulfoxide and propanal were all purchased from Sigma (St. Louis, MO, USA). All other reagents were analytical reagents from Sinopharm Chemical Reagent Co., Ltd. (Shanghai, China). Curcumin analogues 1–30 comes from our study group [15].

### 3.2. Measurement of ALDH1 Activity and Data Set

*In vitro* ALDH1 inhibitory assay was performed as follows [9,10,16]. To a reaction mixture containing 1 mM EDTA, 100 mM KCl, 2 mM NAD, 0.1 M sodium phosphate buffer pH 7.4, 1 U/mg baker’s yeast ALDH1 was added by Multifunction microplatereader (Tecan Infinite 200, TECAN Austria GmbH, Männedorf, Switzerland). The reaction was started by the addition of 1 mM propanal in final volume of 300 μL and the optical density (OD) was read at 340 nm at 0 min (just after the addition of the substrate) and reacted for 5 min. The enzyme activity is expressed as OD/min at 25 °C at the pH 7.4. Results are the average of three experiments done in duplicate. The structures of the compounds and their biological data are given in Table 1. IC_50_ (inhibit growth by 50%). The IC_50_ values in units of μM were transformed in pIC_50_ (–log IC_50_). The data was randomly divided into training set (26 samples) and test set (4 samples * carry out external validation).

### 3.3. Molecular Modeling and Alignment

**Figure 7.**
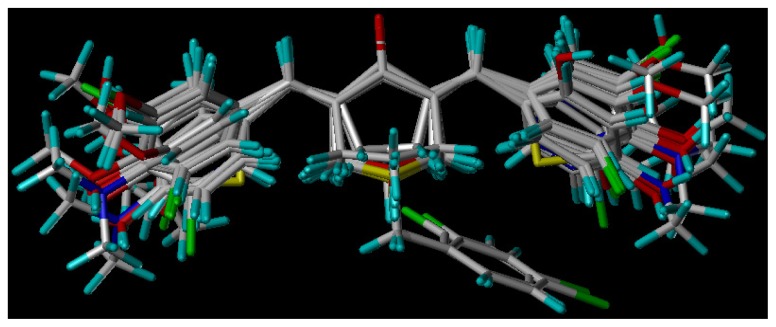
Molecular alignment of the compounds in the training set.

Minimize molecular were performed using SYBYL 8.0 package (Tripos, Shanghai, China). All structures were minimized with the Tripos force field, added the Gasteiger–Hückle. Powel optimize the energy gradient, the maximum times to 2000 times the energy convergence criterion reached 0.005 kcal mol^−1^, and got its 20 small molecule ligand conformations. The most potent CA (compound **6**) was selected as the alignment template molecular. Selecting the appropriate common substructure, the 26 compounds were next aligned. Finally, 26 compounds were aligned to a common substructure of the template using the “align database” command (Figure 7) [17].

### 3.4. 3D-QSAR Models

**Figure 8.**
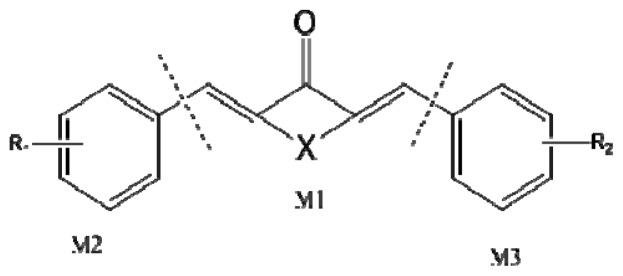
Molecular skeleton region.

3D-QSAR models were constructed by using CoMFA, CoMSIA methods. Parameters of CoMFA and CoMSIA were the default values. The cutoffs value was set 30 kcal/mol. With standard options for scaling of variables, the regression analysis was performed using the “leave-one-out” cross-validation partial least squares method (PLS) and use samples, resulted q^2^ and optimum components [18,19]. The next non-cross-validated model was developed with a no validation PLS analysis. CoMSIA method was performed using steric, electrostatic, hydrophobic, hydrogen-bond donor and hydrogen-bond acceptor descriptors. In this study, the common skeleton diphenyl ketone was split into three pieces by cutting two single bonds (Figure 8).

### 3.5. Molecular Docking

To assess the potent binding conformations and find more insight into the understanding of the interactions of inhibitor, molecular docking analysis was used to the Surflex Dock in SYBYL8.0 [20]. The crystal structure of ALDH1 was retrieved from RCSB Protein Data Bank (PDB: 1BXS) [21]. The crystal structure includes the two dimers, comprised of A, B, C, D four single chain, deleted chain C, B, D and all water molecules. Biopolymer module was then used to repair the crystal structure of the protein termini treatment, fix side chain amides, residues and add charges. By using the Surflex-Docking mode, the potent CAs docking with ALDH1, selected Cys302 as active site and the threshold 0.5, the active pocket formed by computing, the others are the default settings.

## 4. Conclusions

Using *in vitro* assays for inhibitory effect of CAs on ALDH1, compound 6, 7, 8, and 24 exhibited high inhibitory activity. Therefore there is a theory that new ALDH1 inhibitors can be found from the CAs. Meantime, based on the molecular docking results, the 3D-QSAR modeling has developed an understanding of the relationship of molecular structure with ALDH1 inhibitory activity and used a 3D-QSAR model to design a set of novel curcumin analogues with predicted activities. The results indicate that CAs can be a potent ALDH1 inhibitor.

## Figures and Tables

**Table 1.** The structures and bioactivity values of activity of curcumin derivatives.

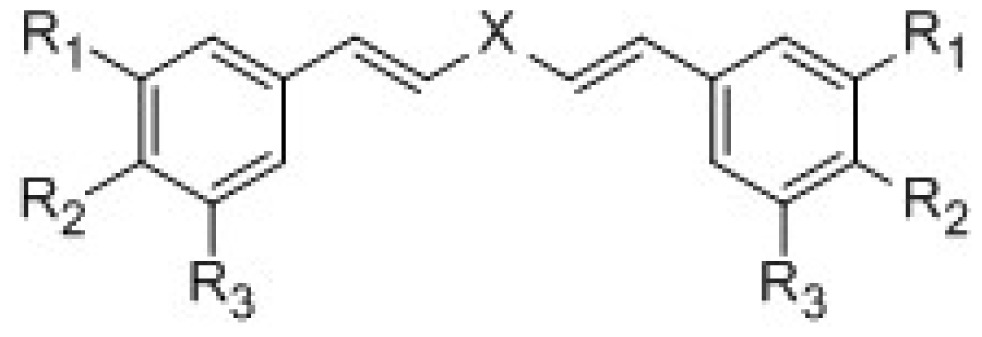						
Compounds	X	R1	R2	R3	IC_50_ μmol/L	pIC_50_
**1**	1-(4-Br-2-F)4-piperidinone	OCH_3_	OH	OCH_3_	31.2	4.51
**2**	1-(4-Br-2-F)4-piperidinone	OH	OH	H	33.5	4.47
**3**	4-piperidinone	OCH_3_	OH	OCH_3_	30.0	4.52
**4**	4-piperidinone	OH	OH	H	39.4	4.40
**5**	Acetone	OH	OH	H	23.6	4.63
**6**	cyclopentanone	OH	OH	H	3.41	5.46
**7**	cyclohexanone	OH	OH	H	6.5	5.20
**8**	tetrahydropyran-4-ones	OH	OH	H	7.9	5.10
**9**	tetrahydrothiopyran-4-one	OH	OH	H	22.7	4.64
**10**	cyclopentanone	H	OH	H	54.2	4.20
**11**	tetrahydropyran-4-ones	H	OH	H	39.7	4.41
**12**	cyclopentanone	OH	H	OH	43.2	4.36
**13**	cyclopentanone	OCH_3_	OH	H	24.2	4.62
**14**	tetrahydrothiopyran-4-one	OCH_3_	OH	H	31.3	4.50
**15**	tetrahydropyran-4-ones	OCH_3_	OH	OCH_3_	16.6	4.78
**16**	tetrahydropyran-4-ones	OCH_3_	OH	F	53.2	4.27
**17**	Acetone	Br	OH	Br	25.8	4.59
**18**	cyclohexanone	Br	OH	Br	17.5	4.76
**19**	tetrahydropyran-4-ones	Br	OH	Br	18.7	4.73
**20**	tetrahydrothiopyran-4-one	Br	OH	Br	17.7	4.75
**21**	tetrahydrothiopyran-4-one	OCH_3_	OCH_3_	OCH_3_	63.1	4.20
**22**	cyclohexanone	H	N(CH_3_)_2_	H	73.5	4.13
**23**	tetrahydrothiopyran-4-one	H	N(CH_3_)_2_	H	69.4	4.16
**24**	cyclohexanone	H	Br	H	8.20	5.08

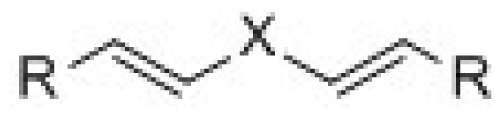						
**Compounds**	**X**		**R**		**IC_50_** **μmol/L**	**pIC_50_**

**25**	Cyclopentanone		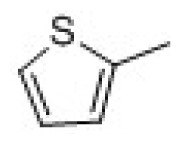		30.1	4.53
**26**	tetrahydrothiopyran-4-one		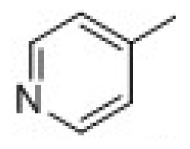		10.6	4.97
**27**	cyclohexanone		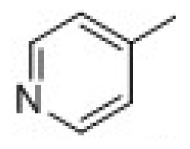		63.2	4.22
**28**	Cyclohexanone		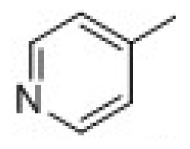		73.4	4.13
**29**	Cyclopentanone		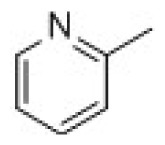		13.7.	4.86
**30**	Cyclohexanone		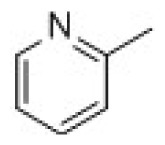		18.4	4.74
Curcumin					36.9	4.43
Disufiram					2.91	5.54

**Table 2.** Summary of the partial-least-squares for the CoMFA/CoMSIA models.

Statistical	*q*^2^	*N*	*r*^2^	*SEE*	*F*	Field Contribution				

						S	E	H	D	A
CoMFA	0.606	9	0.999	0.011	2577.847	0.552	0.448			
CoMFA	0.597	8	0.998	0.018	990.030	0.554	0.446			
SE	0.608	9	0.999	0.082	1924.926	0.386	0.614			
SHE	0.434	6	0.945	0.045	93.12	0.294	0.420	0.287		
SED	0.56	6	0.987	0.031	210.105	0.287	0.3785		0.338	
SEA	0.443	3	0.848	0.134	40.891	0.27	0.388			0.342
SEHD	0.477	3	0.902	0.107	67.688	0.219	0.305	0.182	0.293	
SEDA	0.484	5	0.968	0.064	121.317	0.207	0.295		0.301	0.197
SEHA	0.383	3	0.862	0.128	45.79	0.207	0.319	0.196		0.278
SEHDA	0.421	4	0.945	0.083	89.839	0.166	0.269	0.139	0.247	0.179

*q^2^*, Crossvalidated correlation coefficient using leave-one-out procedure; *N*, optimal number of principal components; *r^2^*, non cross validated correlation coefficient; *F*: F-test value; Steric (S) and Electrostatic (E) field from CoMFA; Steric (S), Eectrostatic (E), Hydrophobic (H), Donor (D), and Acceptor (A) fields from CoMSIA.

**Table 3.** (CoMFA)/(CoMSIA) predicted activity (pIC_50_) of compounds.

Compounds	Actual	CoMFA		CoMSIA	
	
		Predicted	Residues	Predicted	Residues
**1**	4.51	4.533	−0.023	4.540	−0.030
**2**	4.47	4.466	0.004	4.431	0.039
**3**	4.52	4.503	0.017	4.546	−0.026
**4**	4.40	4.418	−0.018	4.433	−0.033
**5**	4.63	4.704	−0.074	4.630	0.000
**6**	5.34	5.341	−0.001	5.360	−0.020
**7**	5.20	5.107	0.093	5.109	0.091
**8**	5.10	5.095	0.005	5.102	−0.002
9*	4.64	4.763	−0.123	4.894	−0.254
10*	4.20	4.557	−0.357	4.404	−0.204
**11**	4.41	4.503	−0.093	4.542	−0.132
**12**	4.36	4.318	0.042	4.333	0.027
**13**	4.62	4.674	−0.054	4.528	0.092
**14**	4.50	4.483	0.017	4.533	−0.033
15*	4.78	4.556	0.224	4.919	−0.139
**16**	4.27	4.307	−0.037	4.277	−0.007
**17**	4.59	4.632	−0.042	4.598	−0.008
**18**	4.76	4.825	−0.065	4.754	0.006
**19**	4.73	4.672	0.058	4.733	−0.003
**20**	4.75	4.705	0.045	4.683	0.067
**21**	4.20	4.138	0.062	4.804	−0.604
**22**	4.13	4.154	−0.024	4.133	−0.003
**23**	4.16	4.152	0.008	4.124	0.036
24*	5.08	4.965	0.115	4.746	0.334
**25**	4.53	4.540	−0.01	4.646	−0.116
**26**	4.97	4.885	0.085	4.685	0.285
**27**	4.22	4.199	0.021	4.202	0.018
**28**	4.13	4.103	0.027	4.131	−0.001
**29**	4.86	4.825	0.035	4.885	−0.025
**30**	4.74	4.709	0.031	4.808	−0.068

* Test set.

**Table 4.** Designed molecules and predicted pIC_50_ values of ALDH1 inhibitors.

(a)			(b)			(c)			

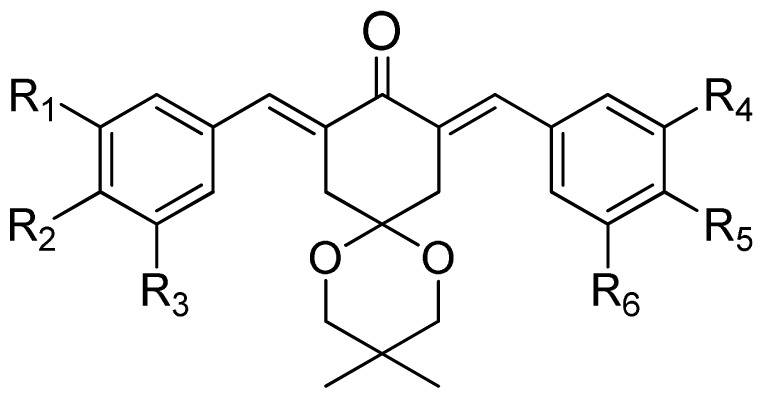			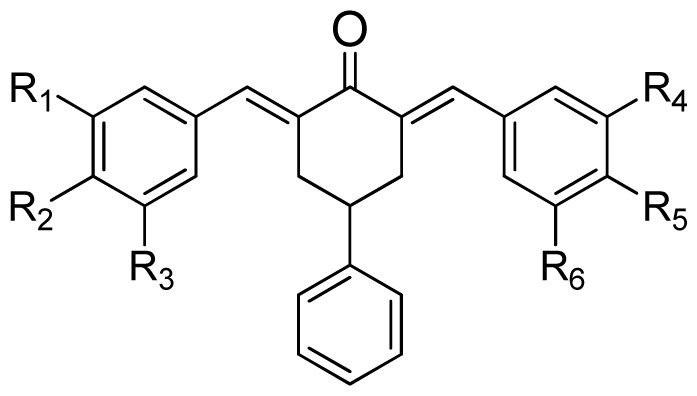			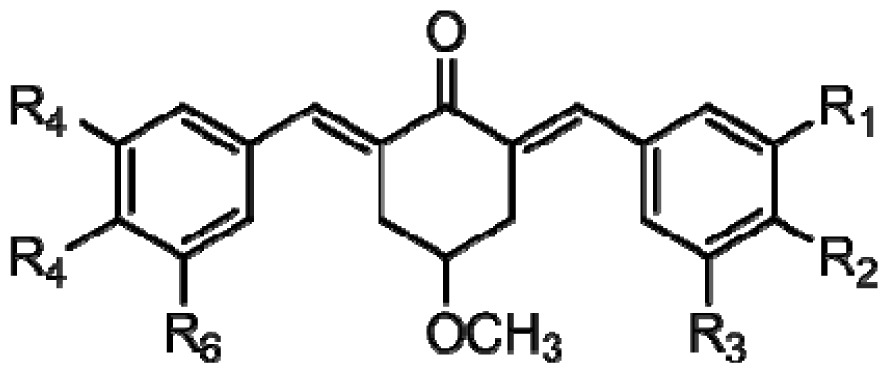			

Compound	Tail	R_1_	R_2_	R_3_	R_4_	R_5_	R_6_	Predict pIC_50_	

								CoMFA	CoMSIA
**1**	a	H	OH	H	H	OCH_3_	H	5.016	4.858
**2**	a	H	OH	H	H	OCH_3_	OCH_3_	5.868	6.124
**3**	a	H	OH	H	OCH_3_	OCH_3_	OCH_3_	5.997	6.077
**4**	a	OH	OH	H	H	OCH_3_	H	6.173	5.766
**5**	a	OH	OH	H	OCH_3_	OCH_3_	H	5.815	6.096
**6**	a	OH	OH	H	OCH_3_	OCH_3_	OCH_3_	5.689	5.966
**7**	a	H	Br	H	H	OCH_3_	H	5.542	6.079
**8**	a	H	Br	H	OCH_3_	OCH_3_	H	5.689	5.993
**9**	a	H	Br	H	OCH_3_	OCH_3_	OCH_3_	5.257	5.857
**10**	b	H	OH	H	H	OCH_3_	H	5.687	6.100
**11**	b	H	OH	H	H	OCH_3_	OCH_3_	5.974	6.240
**12**	b	H	OH	H	OCH_3_	OCH_3_	OCH_3_	5.370	5.725
**13**	b	OH	OH	H	H	OCH_3_	H	6.062	6.127
**14**	b	OH	OH	H	OCH_3_	OCH_3_	H	6.034	6.332
**15**	b	OH	OH	H	OCH_3_	OCH_3_	OCH_3_	5.141	5.739
**16**	b	H	Br	H	H	OCH_3_	H	6.116	6.031
**17**	b	H	Br	H	OCH_3_	OCH_3_	H	6.061	5.991
**18**	b	H	Br	H	OCH_3_	OCH_3_	OCH_3_	5.089	5.859
**19**	c	H	OH	H	H	OCH_3_	H	4.800	4.785
**20**	c	H	OH	H	H	OCH_3_	OCH_3_	6.109	6.199
**21**	c	H	OH	H	OCH_3_	OCH_3_	OCH_3_	5.961	5.926
**22**	c	OH	OH	H	H	OCH_3_	H	6.097	6.077
**23**	c	OH	OH	H	OCH_3_	OCH_3_	H	6.062	5.914
**24**	c	OH	OH	H	OCH_3_	OCH_3_	OCH_3_	5.753	5.914
**25**	c	H	Br	H	H	OCH_3_	H	6.168	6.128
**26**	c	H	Br	H	OCH_3_	OCH_3_	H	6.104	6.253
**27**	c	H	Br	H	OCH_3_	OCH_3_	OCH_3_	6.028	5.842
